# Gemcitabine, oxaliplatin and 5-FU in advanced bile duct and gallbladder carcinoma: two parallel, multicentre phase-II trials

**DOI:** 10.1038/sj.bjc.6605377

**Published:** 2009-11-10

**Authors:** A D Wagner, P Buechner-Steudel, M Moehler, H Schmalenberg, R Behrens, J Fahlke, A Wein, S Behl, O Kuss, G Kleber, W E Fleig

**Affiliations:** 1 First Department of Medicine, University Hospital, Halle 06097 (Saale), Germany; 2 First Department of Medicine, University Hospital, Mainz 55131, Germany; 3 Department of Medicine II, University Hospital, Jena 07740, Germany; 4 Gastroenterology Practice, Halle 06108 (Saale), Germany; 5 Department of Visceral Surgery, University Hospital, Magdeburg 39120, Germany; 6 First Department of Medicine, University Hospital, Erlangen 91054, Germany; 7 Institute of Medical Epidemiology, Biostatistics, and Informatics, Martin-Luther-University Halle-Wittenberg, Halle 06097 (Saale), Germany

**Keywords:** bile duct cancer, gallbladder cancer, gemcitabine, oxaliplatin, 5-FU

## Abstract

**Background:**

Gemcitabine, oxaliplatin and 5-fluorouracil (5-FU) are active in biliary tract cancer and have a potentially synergistic mode of action and non-overlapping toxicity. The objective of these trials was to determine response, survival and toxicity separately in patients with bile duct cancer (BDC) and gallbladder cancer (GBC) treated with gemcitabine/oxaliplatin/5-FU chemotherapy.

**Methods:**

Eligible patients with histologically proven, advanced or metastatic BDC (*n*=37) or GBC (*n*=35) were treated with gemcitabine (900 mg m^−2^ over 30 min), oxaliplatin (65 mg m^−2^) and 5-FU (1500 mg m^−2^ over 24 h) on days 1 and 8 of a 21-day cycle. Tumour response was the primary outcome measure.

**Results:**

Response rates were 19% (95% CI: 6–32%) and 23% (95% CI: 9–37%) for BDC and GBC, respectively. Median survivals were 10.0 months (95% CI: 8.6–12.4) and 9.9 months (95% CI: 7.5–12.2) for BDC and GBC, respectively, and 1- and 2-year survival rates were 40 and 23% in BDC and 34 and 6% in GBC (intention-to-treat analysis). Major grade III and IV adverse events were neutropenia, thrombocytopenia, elevated bilirubin and anorexia.

**Conclusion:**

Triple-drug chemotherapy achieves comparable results for response and survival to previously reported regimens, but with more toxicity.

Although biliary tract cancer (BTC) is a rare cancer in Europe and North America, there is substantial geographical variation, with high mortality rates in Central and Eastern Europe, Japan, India, Korea and Shanghai (China). The highest rates of up to 16.6/100 000 women are seen in Chile (Randi *et al*, 2009). Biliary tract cancer includes gallbladder cancer (GBC) and bile duct cancer (BDC). Bile duct cancer may be further subdivided into intrahepatic cholangiocarcinoma and adenocarcinoma of the extrahepatic bile ducts, which includes Klatskin's tumours at the liver hilus. Unfortunately, fewer than 10% of all patients are candidates for curative resection, and relapse rates remain high after surgery (Hezel and Zhu, 2008). Thus, systemic chemotherapy is given to the majority of patients.

Compared with best supportive care, fluoropyrimidine-based combination chemotherapy has been shown in a randomised trial to improve survival and quality of life (QoL) in bilio-pancreatic cancer (Glimelius *et al*, 1996).

Bile duct cancer is a heterogeneous group with increasing evidence of differences between BDC and GBC at both clinical (Yonemoto *et al*, 2007) and molecular levels (Jarnagin *et al*, 2006). A recently published, pooled analysis of 104 chemotherapy trials in BTC identified response rates of 34.4 and 20.2%, respectively, in patients with GBC and BDC (Eckel and Schmid, 2007). Despite the significantly better response rate in GBC, patients with GBC had a shorter median overall survival (7.2 months) than those with BDC (9.3 months; *P*=0.048). This observation was confirmed by a large (*n*=413), retrospective analysis from Japan reporting a median survival of 8.4 months (95% CI: 5.5–11.2) for intrahepatic cholangiocarcinoma, 10.2 months (95% CI: 5.4–13.7) for extrahepatic BDC and 6.5 months (95% CI: 5.3–8.0) for GBC (Yonemoto *et al*, 2007). In addition, several individual trials including patients with both BDC and GBC have reported clinically important differences between BDC and GBC, ranging from 2.7 to 12.4 months (Sanz-Altamira *et al*, 1998; Patt *et al*, 2001; Alberts *et al*, 2005; Knox *et al*, 2005; Kobayashi *et al*, 2006) in median overall survival, with constantly better results for BDC patients. In a randomised phase II trial, which compared a combination of 5-fluorouracil (5-FU)/cisplatin chemotherapy with single-agent 5-FU chemotherapy in BTC, the response rates and median overall survival tended to be superior with the doublet (18.5 *vs* 7.1% and 8.0 *vs* 5.0 months, respectively); however, the differences were not statistically significant (Ducreux *et al*, 2005). Significantly better response rates (24 *vs* 15%) and survival (8.3 *vs* 11.7 months, *P*=0.002) for the gemcitabine/cisplatin combination – as compared with single-agent gemcitabine – have been reported in the recently presented landmark trial UK ABC-02, which is the largest randomised study in BTC to date (Valle *et al*, 2009). Although results from randomised trials comparing the combination of gemcitabine and 5-FU in BTC are lacking, the available data for the combination of gemcitabine and fluoropyrimidines in pancreatic cancer are controversial. Although the gemcitabine/5-FU combination has no advantage in survival when compared with gemcitabine alone (Riess *et al*, 2005), a randomised phase III trial comparing gemcitabine/capecitabine *vs* gemcitabine alone showed a significant survival benefit for the two-drug combination (HR for survival 0.8; 95% CI: 0.65–0.98; *P*=0.026) (Cunningham *et al*, 2005). Whether treatment results for biliary cancer might be further improved using three-drug combinations is still unclear. On the basis of the evidence of their activity in BTC (Eckel and Schmid, 2007), their non-overlapping toxicity profiles and a potentially synergistic mode of action (Peters *et al*, 1995; Faivre *et al*, 1999), we investigated the efficacy and safety of the triplet combination of gemcitabine, oxaliplatin and infusional 5-FU (GemFOx) in patients with locally advanced or metastatic BDC and GBC.

Separation of the patients into two parallel but separate phase II trials for BDC and GBC was motivated by both, their different prognosis and potentially different response to chemotherapy.

## Patients and methods

### Eligibility criteria

Eligible patients met the following criteria: histologically confirmed, unresectable, locally advanced (UICC stage III, T_1–4_ and N_1_), metastatic (UICC stage IV, T_1–4_, N_1_ and M_1_) or recurring (after resection) adenocarcinoma of the bile ducts (BDC study) or gallbladder (GBC study); bidimensionally measurable tumour lesion; age 18–75 years; ECOG status 0–1; estimated life expectancy of ⩾3 months; and adequate bone marrow function (white blood cell count ⩾3.5 × 10^9^/l, platelets ⩾100 × 10^9^/l). Adjuvant chemotherapy or radiation therapy was permitted if it had been terminated at least 6 months before the inclusion in this trial. Exclusion criteria were borderline tumours, serious comorbid conditions, bilirubin ⩾1.5 times the upper limit of normal (ULN) despite adequate endoscopic biliary drainage, alanine amino transaminase (ALT) and aspartate amino transaminase (AST) levels >5 and 2.5 times, respectively, the ULN in patients with and without liver metastases, creatinine exceeding 200 *μ*mol l^−1^, pregnancy and lactation, earlier chemotherapy, suspected cerebral metastases and peripheral sensory neuropathy. The studies were conducted in accordance with the Declaration of Helsinki Principles and Good Clinical Practice guidelines. Supportive care measures such as antibiotic therapy and administration of haematopoietic growth factors were permitted and used at the discretion of the investigator. Written informed consent was obtained from all patients before entering the study. The Ethics Committee of the Medical Faculty (Martin-Luther-University Halle-Wittenberg, Germany) and the responsible ethics committees for the participating institutions had approved the protocol.

### Study design and treatment

These single-arm, multicentre phase II studies were conducted in parallel at the same institutions. The study protocol was approved and supported by the Arbeitsgemeinschaft Internistische Onkologie (AIO). Similar to our parallel phase II study in pancreatic cancer (Wagner *et al*, 2007), patients were treated with gemcitabine (900 mg m^−2^ as a 30-min infusion), followed by oxaliplatin (65 mg m^−2^ as a 2-h infusion) and 5-FU (1500 mg m^−2^ without folinic acid as a 24-h continuous infusion), all on days 1 and 8 of a 21-day schedule. Treatment was terminated on disease progression or unacceptable toxicity, or at the patient's or investigator's request. Drug doses were modified on the basis of blood counts taken before each administration to maintain a tolerable safety profile. Treatment was interrupted as soon as leukopenia or diarrhoea of >grade I or any other toxicity (except alopecia) of >grade II was observed. If time to recovery from any toxicity was more than 1 week, subsequent doses were reduced to 80%. In case of sensory neuropathy lasting longer than 7 days, oxaliplatin was administered at 80% of the initial dose in subsequent cycles. When functional impairment attributed to sensory neuropathy was not relieved at the time of the next scheduled administration, treatment with oxaliplatin was withheld until recovery.

### Assessment

Pretreatment evaluations consisted of a complete medical history, physical examination, assessment of ECOG status, blood count, blood chemistry including CA 19-9 and urine analysis. Tumour measurements were performed with either computerised tomography or magnetic resonance imaging every 6 weeks, with tumour responses classified according to the WHO criteria (Miller *et al*, 1981). Blood counts were obtained before each chemotherapy administration, and serum chemistry and urine analysis were carried out before each new cycle. Toxicity was classified according to the NCI-CTC (version 2.0). Quality of life was assessed before therapy and after every two cycles using the FACT-Hep questionnaire (Heffernan *et al*, 2002). After discontinuation of treatment, follow-up was scheduled every 3 months.

### Statistical methods and analysis

According to the study protocol, three populations for analysis were defined as follows:

1. *Safety population*: All patients who received the study medication at least once.
2. *Intention-to-treat (ITT) population*: All eligible patients who received the study medication at least once.
3. *Per-protocol (PP) population*: All patients who completed at least two cycles of chemotherapy.

Objective response rate as the primary end point of both studies was assessed in both the PP and ITT populations. The secondary end points – median overall survival, time to progression (TTP) and 1- and 2-year overall survival rates – were analysed in the ITT population only. On the basis of the results published for single-agent therapy with gemcitabine (Valle *et al*, 2009), we predefined overall response rates of 10 and 30% in the PP population as being clinically irrelevant and relevant, respectively. The sample size necessary for the primary end point was calculated accepting a type I error of 5% and a test power of 90%. This resulted in a target enrolment of 35 response-evaluable patients in each study. Time to progression and survival were analysed using standard methods of survival analysis. Survival function was estimated by the Kaplan–Meier method (Kaplan and Meier, 1958). Response rates, median survival times and survival rates are given with 95% CIs.

## Results

### Patients and treatment

**Figure 1:**
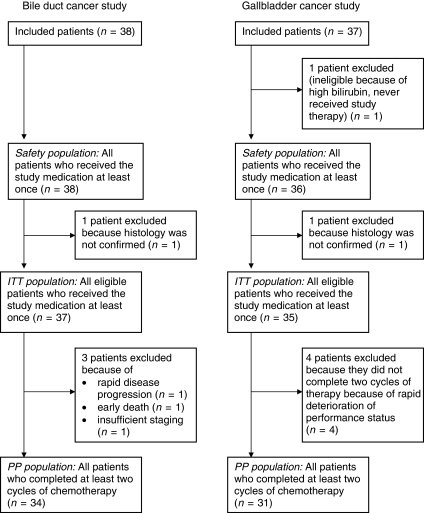
Diagram of patient flow.

A total of 38 patients with BDC and 37 patients with GBC were enrolled between February 2002 and October 2004. Follow-up was performed until death or up to a maximum of 60 months. Patient flow is depicted in Figure 1. Baseline demographic and clinical characteristics of the ITT and PP populations are summarised in Table 1. In the BDC study, a total of 299 cycles of study therapy were started and 280 cycles completed. The median number of cycles per patient was 6.0 (range: 1–28). In the GBC study, a total of 274 treatment cycles were started and 265 completed. The median number of cycles per patient was 8 (range: 0–18).

### Efficacy

**Figure 2:**
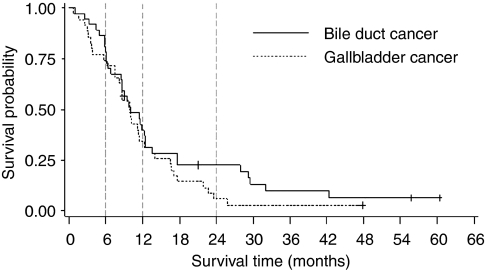
Overall survival (Kaplan–Meier) for patients with gallbladder cancer (*n*=35) and bile duct cancer (*n*=37) (ITT population). +, censored observations.

Objective response rates in both the PP and ITT populations are presented in Table 2. No patient in either study showed a complete response and none of the patients with a partial response qualified for secondary resection. The Kaplan–Meier curves for overall survival of the ITT populations in both trials are depicted in Figure 2.

#### BDC trial

On ITT analysis, median overall survival and time to progression were 10.0 (95% CI: 8.6–12.4) and 6.2 (95% CI: 5.1–11.5) months, respectively. One- and 2-year overall survival rates were 40% (95% CI: 25–56%) and 23% (95% CI: 11–38%), respectively.

#### GBC trial

On ITT analysis, median overall survival and time to progression were 9.9 (95% CI: 7.5–12.2) and 5.7 (95% CI: 3.1–8.1) months, respectively. One- and 2-year overall survival rates were 34% (95% CI: 20–51%) and 6% (95% CI: 1–16%), respectively.

**Figure 3:**
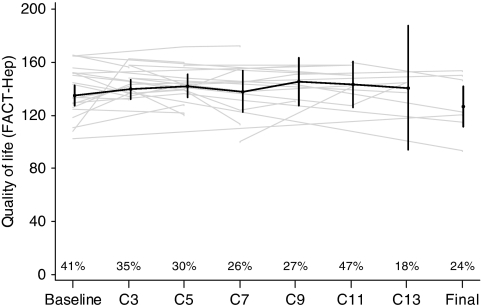
Quality of life (QoL), as measured by the FACT-Hep global score. Given are the individual (grey) and mean (black) global QoL scores (with 95% confidence interval), as measured by the FACT-Hep questionnaire. High scores represent a high QoL with the maximum achievable value being 180, the minimum value 0. Quality of life was evaluated at baseline, before cycles 3–13 and at the final evaluation. Percentage values within the figure give the fraction of questionnaires divided by the number of patients still in the study at the respective time point.

The pooled results for QoL from both studies, as measured by the FACT-Hep questionnaire, are depicted in Figure 3. Overall, the FACT-Hep global score in those patients who completed the questionnaires remained stable over the treatment period. However, these data should be interpreted with great caution because of a significant number of missing questionnaires. Bias attributed to the selective return of questionnaires from patients in good performance status cannot be excluded for this reason.

### Adverse events

The most common adverse events, rated by the attending physician as NCI-CTC grade I–IV, are reported in Table 3. Treatment was well tolerated in the majority of patients. Most patients in both trials discontinued study therapy because of disease progression or deteriorating performance status. There were no treatment-related deaths.

#### BDC trial

A total of 25 serious adverse events occurred; of these, 12 were obstructive jaundice requiring endoscopic treatment. One patient each experienced deep venous thrombosis and pulmonary embolism. All serious adverse events except one were regarded as unrelated to therapy by the investigators. One patient with a history of cardiomyopathy suffered sudden death 1 day after the administration of chemotherapy. Whether this event was related to the underlying disease, 5-FU-associated cardio toxicity or both is not clear. A total of 21 hospitalisations were necessary in BDC patients; among these, 9 were attributable to febrile infections, mostly cholangitis (8 patients). One patient in this group had febrile neutropenia.

Chemotherapy was administered as planned for 55.3% of scheduled treatments. Dosage had to be reduced for 20% or treatment deferred for 15%, or both for 7.8%, of scheduled administrations. In one patient, therapy was discontinued because of an allergic reaction to oxaliplatin.

#### GBC trial

A total of 19 serious adverse events occurred. Only one of these (dysphagia and sustained vomiting with onset soon after the administration of chemotherapy) was considered as possibly related to the study therapy by the investigator. Most other serious adverse events were rated as associated with the underlying disease. There were no cardiac events or sudden deaths. Chemotherapy was administered as planned for 46.7% of scheduled treatments, was delayed for 17.9%, required dose reduction for 18.9% or required both deferral and dose reduction for 13.4%. One patient discontinued study therapy because of oxaliplatin-induced polyneuropathy. A total of 17 hospitalisations occurred during and in the first 30 days after chemotherapy in GBC patients. Among these, three were attributed to febrile infections, among which two were febrile neutropenia.

## Discussion

On the basis of favourable results of studies using two-drug combinations including gemcitabine, oxaliplatin and fluoropyrimidines (instead of 5-FU) (Alberts *et al*, 2005; Knox *et al*, 2005; Nehls *et al*, 2008), our trials were conducted to establish the efficacy and safety of the triplet GemFOx chemotherapy in patients with BDC and GBC. Taken together, these multicentre phase II studies, which include a total of 72 patients, represent one of the largest published series evaluating a single combination chemotherapy regimen in BTC. In addition, follow-up (up to 60 months) in this trial was longer than in most other studies. As results from randomised studies of chemotherapy in BTC are limited to two phase III trials (*n*=54) (Rao *et al*, 2005; Valle *et al*, 2009) and a small number of phase II trials (*n*=22–86 patients) (Kornek *et al*, 2004; Ducreux *et al*, 2005; Rao *et al*, 2005; Ciuleanu *et al*, 2007), treatment decisions in these tumours have to also consider the results of single-arm trials.

Both BDC and GBC are a clinically heterogeneous group of cancers. Increasing evidence that these differences are also seen at the molecular levels (Jarnagin *et al*, 2006) lends further support to the notion that ‘gallbladder cancer is a different disease that needs individual trials’ (Gallardo *et al*, 2005). Despite their heterogeneity, the majority of previously published trials include both BDC and GBC. In contrast, we evaluated – for the first time – the same combination chemotherapy regimen separately and with adequate power in patients with BDC and GBC. Although our trials are inevitably limited by the single-arm study design, they provide important and new clinical information regarding the efficacy and toxicity of the triplet chemotherapy combination under investigation.

In our BDC trial, although the objective response rate of 19% and the median overall survival of 10 months compare favourably with the results obtained with single-agent 5-FU or gemcitabine (Ducreux *et al*, 2005; Valle *et al*, 2009), the hypothesis that GemFOx might further improve on the results achieved with modern two-drug combinations (André *et al*, 2004, 2008; Alberts *et al*, 2005; Knox *et al*, 2005; Kim *et al*, 2006) was not confirmed. Nehls *et al* (2008) recently observed that adenocarcinoma of extrahepatic bile ducts, including Klatskin's tumours of the liver hilus, seems to have a better prognosis and response to chemotherapy than does intrahepatic cholangiocarcinoma. Furthermore, a differential expression of molecular targets such as HER-2 has been shown recently in intrahepatic and extrahepatic BDC (Yoshikawa *et al*, 2008). Therefore, possible variations in the relative proportions of intrahepatic and extrahepatic BDC might be a confounding factor in previously published studies, rendering conclusions regarding the efficacy of particular chemotherapy regimens difficult. Although we had insufficient data to retrospectively stratify our patients with respect to BDC subtype, and sound published epidemiological data are lacking, an unpublished retrospective 3-year (1 January 2006 to 31 December 2008) histopathological series of 839 unselected, consecutive cases of BDC in Germany suggests that ∼70% of these tumours are adenocarcinomas of the extrahepatic bile ducts (A Tannapfel, personal communication, January 2009). Future studies should address and clarify this issue by prospectively stratifying for the intrahepatic and extrahepatic type of BDC.

For GBC, owing to the even more limited data, the question of whether our triplet chemotherapy combination is superior to the single-agent or two-drug combination regimens tested in previous trials is more difficult to answer. Only 12 single-arm phase II and no phase III studies in patients with GBC were identified in a recent systematic search (Eckel and Schmid, 2007). Separate results for GBC were reported in 18 further studies with a mixed BTC population (F Eckel, personal communication, April 2008). We updated this search in October 2008 and identified only one additional abstract describing a prospective chemotherapy trial in GBC (Gallardo *et al*, 2008). Overall survival in the larger series (⩾40 patients) was 5.7 (Chatni *et al*, 2008), 7.0 (Reyes-Vidal *et al*, 2003), 7.4 (Misra *et al*, 2005) and 9.0 months (Gallardo *et al*, 2008). Published results for GBC of all multicentre series, which included more than 20 patients, are available as Supplementary Online Material. Compared with these series, the median overall survival of 9.9 months and 1-year overall survival rate of 36% observed in our trial are encouraging. However, these results require confirmation in a randomised study.

Intensified chemotherapy using a three-drug combination might eventually allow for secondary resection of initially unresectable disease. In fact, none of the patients in our trials qualified for secondary resection, and we consider it unlikely that other currently available chemotherapy combinations will perform better. Thus, achieving secondary resectability appears to be an unrealistic goal for chemotherapy regimens available at present in patients with BTC.

The second major goal of our trials was to define the toxicity of this triplet chemotherapy in the two patient populations: Overall, the toxicity of the GemFOx combination chemotherapy in both patient populations was manageable. However, as expected, the rates of grade III and IV neutropenia (36.9 and 33.4% in patients with BDC and GBC), thrombopenia (18.4 and 36.1% in BDC and GBC) and anorexia (21 and 25.0% in BDC and GBC) in our study were increased when compared with the recently presented data for the gemcitabine/cisplatin combination in BTC (grade III/IV neutropenia 22.6%, thrombopenia 8.2% and anorexia 1.9%) (Valle *et al*, 2009). However, events such as febrile neutropenia (2.6 and 5.6% in BDC and GBC, respectively) or grade III/IV bleeding were low, and there were no treatment-related deaths. As expected, the risk of cholangitis was more important in patients with BDC compared with those with GBC. In contrast to our trial in pancreatic cancer (Wagner *et al*, 2007), the incidence of cardiovascular events was unremarkable.

In conclusion, a GemFOx triplet chemotherapy regimen is feasible in patients with advanced BTC, although the toxicity is increased compared with doublets. Although for patients with BDC the response rates and median overall survival in our trial do not exceed the results reported for the use of chemotherapy doublets, median overall survival for GBC compares favourably with most other published trials. However, the number of patients included in this trial is too small to draw definitive conclusions about the balance between benefit and toxicity of this three-drug combination chemotherapy regimen in BTC. Future drug development in BTC must account for the heterogeneity of BTC. The evolving understanding of the biology of these tumours confirms the existence of differences between GBC, as well as intrahepatic and extrahepatic BDC, at the molecular level. More importantly, it provides a basis for the rational use of targeted therapies that are currently under clinical evaluation. Whether tumour response is an appropriate primary end point for future clinical trials in biliary neoplasms is still open to question (Gores *et al*, 2004). Overall survival and patient-reported QoL have the advantage of being direct indicators of patient benefit.

## Figures and Tables

**Table 1:** Baseline characteristics

	**BDC study**		**GBC study**	
	**ITT population**	**PP population^a^**	**ITT population**	**PP population^b^**
Number of patients	37	34	35	31
				
*Age (years)*				
Median	61.5	61.7	64.1	63.9
Range	36.1–75.9	38.3–74.8	42.6–79.8	42.6–79.8
				
*Gender, n (%)*				
Male	17 (46)	16 (47)	10 (29)	9 (29)
Female	20 (54)	18 (53)	25 (71)	22 (71)
				
*ECOG status, n (%)*				
Median	0	0	0	0
0	24 (65)	24 (71)	25 (71)	22 (71)
1	12 (32)	10 (29)	10 (29)	9 (29)
				
*Disease status, n (%)*				
Locally advanced	8 (22)	7 (21)	3 (9)	3 (10)
Metastatic	29 (78)	27 (79)	32 (91)	28 (90)
				
*Number of organs with metastases, n (%)*				
0	4 (11)	4 (12)	1 (3)	1 (3)
1	17 (46)	16 (47)	13 (37)	12 (39)
2	9 (24)	7 (21)	16 (46)	14 (45)
3	5 (14)	5 (15)	2 (6)	1 (3)
>3	2 (5)	2 (6)	3 (9)	3 (10)
				
Recurrence after prior surgical resection, *n* (%)	15 (41)	14 (41)	10 (29)	9 (29)

Abbreviations: BDC=bile duct cancer; GBC=gallbladder cancer; ITT population=intention-to-treat population; PP population=per-protocol population. The definitions of both populations are provided in the section ‘Statistical methods and analysis’. The patient flow in both populations is depicted in Figure 1.

a Two patients were not eligible for the assessment of response according to the protocol because they did not complete two cycles of therapy. One more patient could not be assigned a response category because of insufficient staging.

b Four patients were not eligible for the assessment of response because they did not complete two cycles of therapy due to rapid deterioration of their functional status. Results for the ITT analysis are provided in the text.

**Table 2:** Tumour response (WHO) (Miller *et al*, 1981) (ITT and PP populations)

	**BDC study**		**GBC study**	
	**ITT population Number (%) of evaluable patients (*n*=37)**	**PP population Number (%) of evaluable patients (*n*=34)**	**ITT population Number (%) of evaluable patients (*n*=35)**	**PP population Number (%) of evaluable patients (*n*=31)**
CR	0 (0)	0 (0)	0 (0)	0 (0)
PR	7 (19)	7 (20)	8 (23)	8 (26)
NC	20 (54)	20 (59)	16 (46)	16 (52)
PD	6 (16)	6 (18)	6 (17)	6 (19)
				
Not assessable	4 (11)	1 (3)	5 (14)	1 (3)
				
Overall response (CR+PR)	7 (19)	7 (20)	8 (23)	8 (26)
Disease control (CR+PR+NC)	27 (73)	27 (79)	24 (69)	24 (77)

Abbreviations: BDC=bile duct cancer; CR=complete response; GBC=gallbladder cancer; ITT population=intention-to-treat-population; NC=no change; PD=progressive disease; PR=partial response; PP population=per-protocol-population (both populations have been defined in the text; see ‘Statistical methods and analysis’ section).

**Table 3:** Most frequent grade III/IV adverse effects (NCI-CTC) observed in BDC and GBC trials

	**BDC**				**GBC**			
	**Grade I**	**Grade II**	**Grade III**	**Grade IV**	**Grade I**	**Grade II**	**Grade III**	**Grade IV**
Haemoglobin	18 (47.4)	14 (36.8)	4 (10.5)	2 (5.3)	21 (58.3)	18 (50.0)	2 (5.6)	0 (0)
Leukocytes	21 (55.3)	17 (44.7)	15 (13.2)	0 (0)	26 (72.2)	15 (41.7)	2 (5.6)	1 (2.8)
Neutrophils/granulocytes	15 (39.5)	12 (31.6)	9 (23.7)	5 (13.2)	18 (50.0)	13 (36.1)	10 (27.8)	2 (5.6)
Platelets	21 (55.3)	11 (28.9)	3 (7.9)	4 (10.5)	16 (44.4)	15 (41.7)	10 (27.8)	3 (8.3)
Bilirubin	1 (2.6)	10 (26.3)	7 (18.4)	8 (21.1)	2 (5.6)	10 (27.8)	5 (13.9)	1 (2.8)
AST/ALT	29 (76.3)	16 (42.1)	9 (23.7)	0 (0)	27 (75.0)	10 (27.8)	2 (5.6)	0 (0)
Alkaline phosphatase	26 (68.4)	15 (39.5)	7 (18.4)	0 (0)	24 (66.7)	9 (25.0)	4 (11.1)	0 (0)
Nausea	27 (71.1)	12 (31.6)	1 (2.6)	0 (0)	28 (77.8)	16 (44.4)	3 (8.3)	0 (0)
Diarrhoea	15 (39.5)	2 (5.3)	2 (5.3)	0 (0)	13 (36.1)	6 (16.7)	0 (0)	0 (0)
Anorexia	23 (60.5)	7 (18.4)	6 (15.8)	2 (5.3)	29 (80.6)	16 (44.4)	8 (22.2)	1 (2.8)
Oedema	5 (13.2)	8 (21.1)	6 (15.8)	0 (0)	6 (16.7)	7 (19.4)	3 (8.3)	0 (0)
Dyspnea	3 (7.9)	3 (7.9)	3 (7.9)	1 (2.6)	3 (8.3)	5 (13.9)	3 (8.3)	2 (5.6)
Sensory neuropathy	24 (63.2)	10 (26.3)	3 (7.9)	0 (0)	26 (72.2)	19 (52.8)	7 (19.4)	0 (0)
Fatigue	18 (47.4)	27 (71.1)	3 (7.9)	1 (2.6)	21 (58.3)	24 (66.7)	5 (13.9)	1 (2.8)
Fever	11 (28.9)	12 (31.6)	2 (5.3)	0 (0)	9 (25.0)	10 (27.8)	3 (8.3)	0 (0)
Infection	6 (15.8)	11 (28.9)	3 (7.9)	0 (0)	5 (13.9)	13 (36.1)	3 (8.3)	0 (0)
Febrile neutropenia	0 (0)	0 (0)	1 (2.6)	0 (0)	0 (0)	0 (0)	2 (5.6)	(0)

Abbreviations: ALT=alanine amino transaminase; AST=aspartate amino transaminase; BDC=bile duct cancer; GBC=gallbladder cancer.

Absolute (and relative, in %) number of patients.
