# Supplementary material for: Gemcitabine, oxaliplatin and 5-FU in advanced bile duct and gallbladder carcinoma: two parallel, multicentre phase-II trials
Source: Br J Cancer. 2009 Nov 10;101(11):1846–52. doi: 10.1038/sj.bjc.6605377 (PMC2788250; doi:10.1038/sj.bjc.6605377)
Supplement: Supplementary Online Material [file 6605377x1.doc]

**SUPPLEMENTARY ONLINE MATERIAL**

Treatment results for gallbladder cancer (only multi-center studies of > 20 patients):

| **Regimen (Authors)** | **N** | **ORR**  **(95 % CI)** | **OS (mo)**  **(95 % CI)** | **1-Y-OS** |
| --- | --- | --- | --- | --- |
| Gem/CDDP (Doval *et al,* 2004) | 30 | n. r. | 4.6 (3.2 - 7.1) | 18.6 % |
| Gem/CDDP (Abid *et al,* 2003) | 36 | 39 % | 5.7 |  |
| CDDP/5-FU (Chatni *et al,* 2008) | 65 | 8 % (3 – 16 %) | 5.7 (3.6 – 7.8) | 18.5 % |
| Gem/LOHP (Sharma *et al,* 2006) | 32 | 16 % | 6 | at 10 mo: 12 % |
| Gem/LOHP (Andre *et al,* 2008) | 25 | 17 % | 6.1 | approx. 20 %* |
| Gem/Cape (Knox *et al,* 2005) | 22 |  | 6.6 |  |
| Gem (Gallardo *et al,* 2001) | 26 | 36 % | 6.9 |  |
| Gem/CDDP (Reyes-Vidal *et al,* 2003) | 44 | 48 % | 7.0 (6.0 - 8.5) |  |
| Gem/CDDP (Misra *et al,* 2005) | 40 | 55 % | 7.4 (4.1 - 8.3) |  |
| 5-FU/LV +HU (Gebbia *et al,* 1996) | 30 | 30 % | 8.0 | approx. 20 %* |
| Cape/LOHP (Nehls et al, 2008) | 27 | 30% | 8.0 | approx. 33 %* |
| Gem/CDDP (cohort 2) (Gallardo *et al,* 2008) | 44 | 45 % | 9.0 |  |
| **Gem/LOHP/5-FU (Wagner et al, present series)** | **37** | **26 %** | **9.9** | **34 %** |
| Gem/CDDP (cohort 3) (Gallardo *et al,* 2008) | 32 | 41 % | 10.4 |  |
| 5-FU/FA (Malik *et al,* 2003) | 30 | 7 % | 14.8 | approx. 20 %* |
| Gem/Cape (Cho *et al,* 2005) | 24 | 33 % | 16 (13.8 - 18.3) | 58 % |

**Legend:**

*Abbreviations:* Gem = gemcitabine; CDDP = cisplatin, CVI = continuous venous infusion, cape = capecitabine, LV = leucovorin, FA = folinic acid,

HU = hydroxyurea, Carbo = carboplatin, LOHP = oxaliplatin, IFN = interferon, Doxo = doxorubicin. ORR= objective response rate, 1-Y-OS = one-year-overall survival, n. r. = not reported, approx. = approximately, mo = months, 95 % CI = 95 % confidence interval, * survival rates were read from the Kaplan-Meier- graph.

**Supplementary references (references in the supplementary table which are not quoted in the text of the article):**

Abid, L, Oukkal, M., Berkane, S., Mahfouf, H., Asselah, J., and Bouzid, K (2003) Phase II trial with the gemcitabine and cisplatin combination in the treatment of locally advanced and metastatic gall bladder carcinoma. Proc Am Soc Clin Oncol **22**: (abstr 1302)

Cho JY, Nam JS, Park MS, Yu JS, Paik YH, Lee SJ, Lee DK, Yoon DS (2005) A Phase II study of capecitabine combined with gemcitabine in patients with advanced gallbladder carcinoma. Yonsei Med J **46**: 526-531, doi:200508526

Doval DC, Sekhon JS, Gupta SK, Fuloria J, Shukla VK, Gupta S, Awasthy BS (2004) A phase II study of gemcitabine and cisplatin in chemotherapy-naive, unresectable gall bladder cancer. Br J Cancer **90**: 1516-1520, doi:10.1038/sj.bjc.6601736

Gallardo JO, Rubio B, Fodor M, Orlandi L, Yanez M, Gamargo C, Ahumada M (2001) A phase II study of gemcitabine in gallbladder carcinoma. Ann Oncol **12**: 1403-1406

Gebbia V, Majello E, Testa A, Pezzella G, Giuseppe S, Giotta F, Riccardi F, Fortunato S, Colucci G, Gebbia N (1996) Treatment of advanced adenocarcinomas of the exocrine pancreas and the gallbladder with 5-fluorouracil, high dose levofolinic acid and oral hydroxyurea on a weekly schedule. Results of a multicenter study of the Southern Italy Oncology Group (G.O.I.M.). Cancer **78**: 1300-1307, doi:10.1002/(SICI)1097-0142(19960915)78:6<1300::AID-CNCR19>3.0.CO;2-4

Malik IA, Aziz Z (2003) Prospective evaluation of efficacy and toxicity of 5-fu and folinic acid (Mayo Clinic regimen) in patients with advanced cancer of the gallbladder. Am J Clin Oncol **26**: 124-126, doi:10.1097/01.COC.0000017090.36834.FE

Sharma A, Raina V, Shukla NK, Deo SV (2006) Gemcitabine and oxalipaltin (Gemox) in advanced/unresectable gallbladder cancer: A phase II study. ASCO Annual Meeting Proceedings **24**: (abstract 14027)
